# 521. A phase II, double blind, placebo-controlled, randomized evaluation of the safety and efficacy of tafenoquine in patients with mild-moderate COVID-19 disease

**DOI:** 10.1093/ofid/ofad500.590

**Published:** 2023-11-27

**Authors:** Geoffrey Dow

**Affiliations:** 60 Degrees Pharmaceuticals, INC, Washington, District of Columbia

## Abstract

**Background:**

Tafenoquine is an 8-aminoquinoline antimalarial approved by the US FDA for malaria prevention and is being evaluated in clinical trials for potential use in other disease areas.

**Methods:**

The safety and efficacy of tafenoquine administered as a 200 mg dose once per day on days 1, 2, 3, and 10 was evaluated over a 28-day period in mild-moderate COVID-19 patients. The primary endpoint was Day 14 clinical recovery from COVID-19 symptoms, defined as cough mild or absent, respiratory rate < 24 bpm, and no shortness of breath or fever. Following a successful futility analysis after n = 86 patients out of a target n = 275 were randomized, the study was terminated and unblinded early to facilitate planning for confirmatory studies.

**Results:**

The proportion of patients not recovered on Day 14 was numerically decreased by 27% in the ITT population [8/45 v 10/42 not recovered in the tafenoquine and placebo arms, P = 0.60] and 47% in the PP population [5/42 v 9/41, P = 0.25]. Amongst individuals who recorded responses in an electronic diary at Day 28, all tafenoquine patients were recovered, whereas up to 12% of placebo patients exhibited lingering dyspnea. Time to clinical recovery from COVID-19 symptoms was accelerated in the tafenoquine arm by about 2-2.5 days. There were two COVID-19 related hospitalizations in the placebo arm and one in the tafenoquine arm. Mild, drug related adverse events occurred in 8.4% of individuals in the tafenoquine arm [v 2.4% in the placebo].

**Conclusion:**

Although this trial was underpowered for the primary endpoint due to its early termination, the data are suggestive of a therapeutic benefit associated with tafenoquine administration in outpatients with mild to moderate COVID-19 disease, and larger studies are planned.

**Disclosures:**

**Geoffrey Dow, PhD**, 60 Degrees Pharmaceuticals, INC: Board Member|60 Degrees Pharmaceuticals, INC: Inventor on Company covid + malaria patents|60 Degrees Pharmaceuticals, INC: Ownership Interest

